# Foramen Magnum Decompression for Retro-Odontoid Pseudotumor in an Elderly Patient With Chiari Malformation Type I: A Case Report

**DOI:** 10.7759/cureus.106677

**Published:** 2026-04-08

**Authors:** Ryunosuke Yoshihara, Yasufumi Ohtake, Michiru Katayama, Hirohiko Nakamura

**Affiliations:** 1 Neurological Surgery, Nakamura Memorial Hospital, Sapporo, JPN

**Keywords:** arnold-chairi malformation, artificial dura, foramen magnum lesion, retro-odontoid pseudotumor, syringo-hydromyelia

## Abstract

Chiari malformation type I (CM-I) is uncommon in elderly patients, and surgical experience in this population remains limited. Long-term outcomes after surgery, particularly following prolonged conservative observation, have rarely been reported. We present an 82-year-old woman with CM-I who developed neurological deterioration due to a retro-odontoid pseudotumor (ROP) after two decades of clinical stability. Foramen magnum decompression (FMD) was performed without postoperative complications, resulting in marked neurological improvement. She was discharged, and long-term follow-up demonstrated sustained clinical and radiological recovery. This case highlights that advanced age alone should not preclude surgical intervention in CM-I when progressive neurological decline occurs.

## Introduction

Chiari malformation type I (CM-I) is characterized by caudal herniation of the cerebellar tonsils through the foramen magnum, resulting in compression of the cervicomedullary junction and disturbance of cerebrospinal fluid (CSF) circulation [1]. Although CM-I most commonly presents in young or middle-aged adults, its occurrence in the elderly population is rare, and surgical experience in this age group remains limited [2]. The pathophysiology of CM-I in older patients may differ from that in younger individuals, as age-related degenerative changes at the craniovertebral junction can further impair CSF dynamics and contribute to secondary complications.

Retro-odontoid pseudotumor (ROP) is a non-neoplastic fibrocartilaginous mass located posterior to the odontoid process, which results in myelopathy due to anterior compression of the upper cervical cord [3]. It is typically associated with chronic atlantoaxial instability or rheumatoid arthritis, but has also been reported in cases without overt instability [4]. The coexistence of CM-I and ROP is rare, and the underlying mechanisms remain unclear. Altered CSF flow, chronic mechanical stress, and microinstability at the craniovertebral junction have been proposed as contributing factors. In elderly patients, degenerative changes such as ligamentous hypertrophy and osteophyte formation may further predispose them to the development of ROP.

The optimal surgical management of patients with CM-I associated with ROP remains controversial. Some authors advocate atlantoaxial fixation to eliminate instability and promote regression of the ROP, whereas others have reported favorable outcomes following foramen magnum decompression (FMD) alone, particularly in cases without instability [5-7]. In elderly individuals, the surgical strategy should balance adequate decompression with minimal invasiveness, taking into account bone fragility and comorbidities.

This case is significant as it highlights the potential for late-onset neurological deterioration even after decades of clinical stability in CM-I. By documenting this clinical course and reviewing the literature, we aim to clarify the triggers for such delayed progression and discuss the optimal timing and strategies for surgical intervention in long-standing, stable cases.

## Case presentation

An 82-year-old woman presented with a one-year history of progressive headache, bilateral upper limb pain and numbness, and gait disturbance. Her medical history included dyslipidemia and type 2 diabetes mellitus.

**Figure 1:**
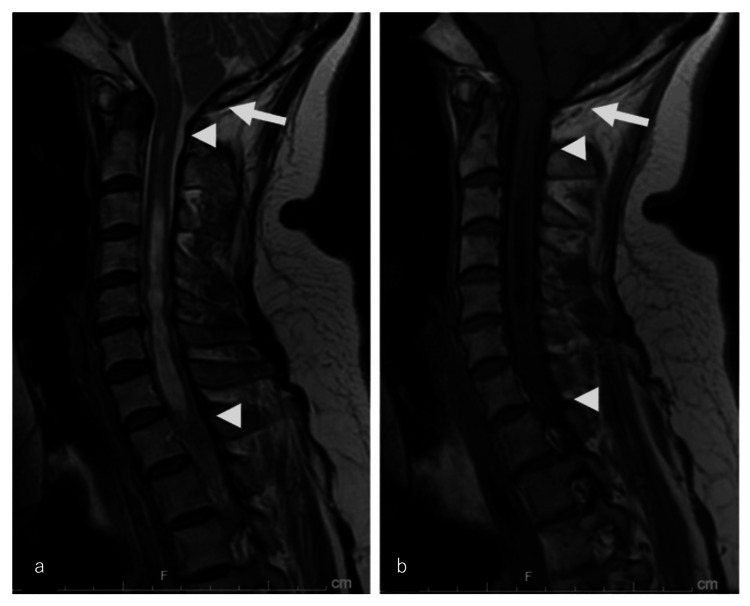
Initial cervical spine MRI obtained 21 years before presentation (a) Sagittal T2-weighted image, (b) Sagittal T1-weighted image. The arrow indicates herniation of the cerebellar tonsils below the foramen magnum. The arrowhead shows a syrinx extending from C2 to T1, appearing hyperintense on T2-weighted images and hypointense on T1-weighted images.

Her notable past medical history included CM-I diagnosed 21 years before presentation. At that time, she experienced recurrent headaches and dizziness, which prompted a neurosurgical consultation. Magnetic resonance imaging (MRI) revealed CM-I with syringomyelia (Figure 1). As her symptoms were mild, conservative management was chosen, and she remained under periodic observation. She remained neurologically stable for nearly two decades.

**Figure 2:**
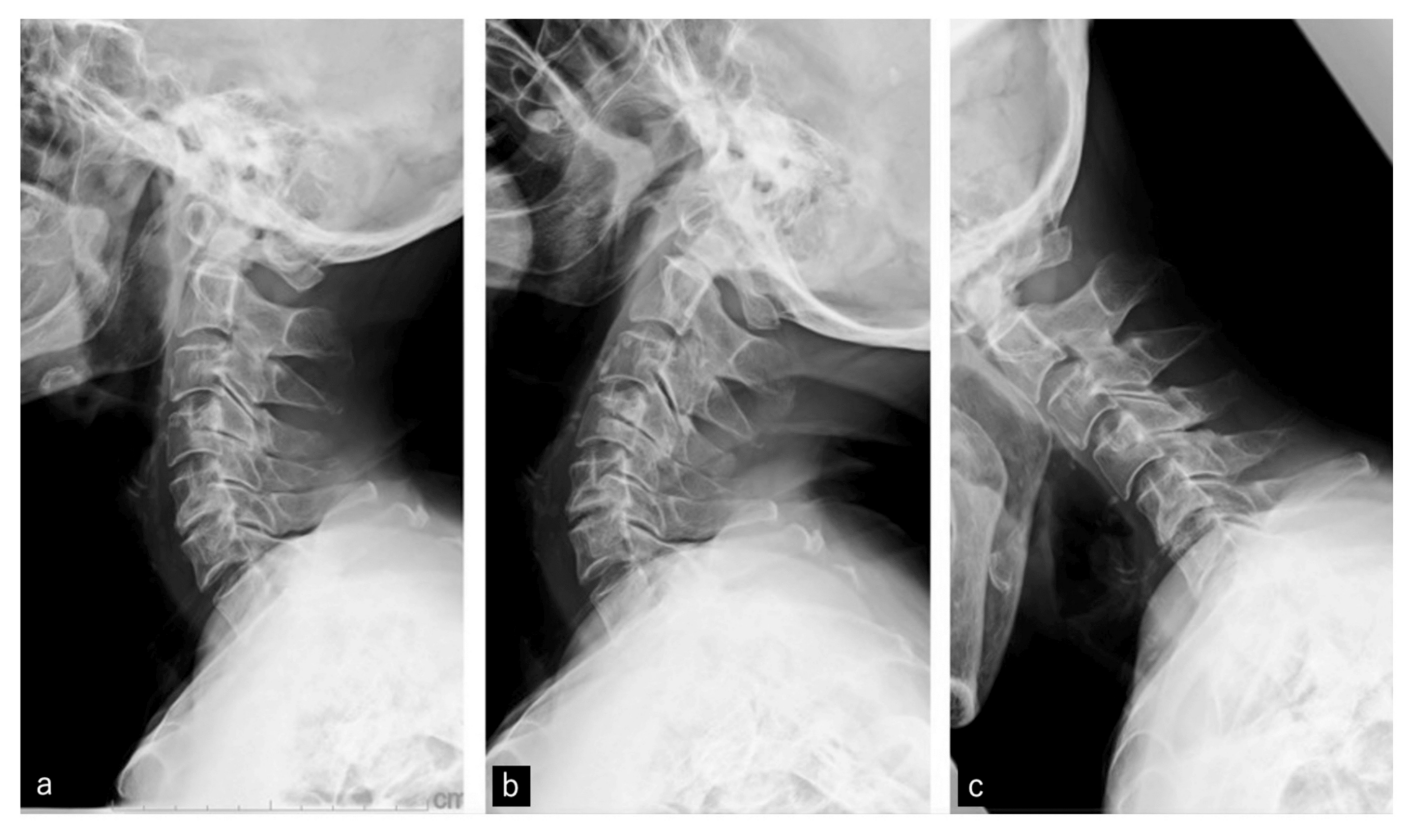
Cervical radiographs in dynamic positions (a) Neutral, (b) extension, (c) flexion. The images confirm the absence of atlantoaxial instability.

The neurological deficit was objectively assessed using the American Spinal Injury Association (ASIA) motor score [8]. Her Upper Extremity Motor Score (UEMS) was 31/50, with notable weakness in wrist extension (C6) and finger function (C8, T1), while the Lower Extremity Motor Score (LEMS) remained relatively preserved at 40/50. Pain was assessed using the numeric rating scale (NRS) for pain severity, and numbness was evaluated using the NRS for numbness severity. Pain and numbness were more pronounced in the left arm, with an NRS score of 8 for pain and 8 for numbness. Intermittent numbness also affected the toes, contributing to gait instability. Based on these findings, the severity of the spinal cord impairment was classified as ASIA Impairment Scale (AIS) Grade D. Dynamic cervical radiography showed no atlantoaxial instability (Figure 2).

**Figure 3:**
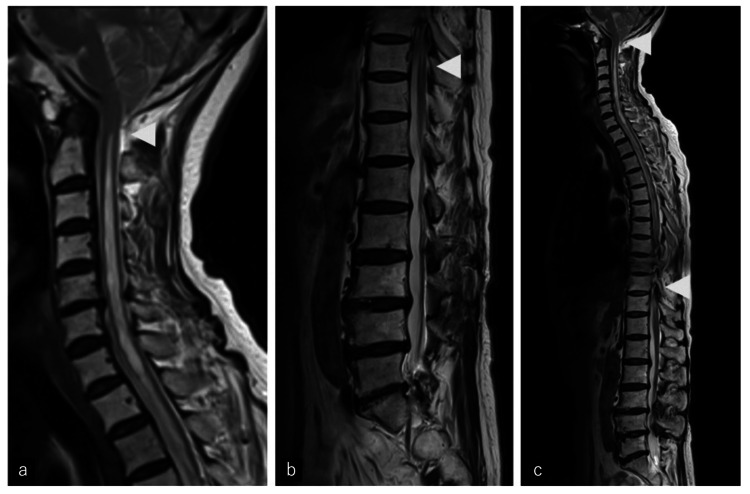
Spinal magnetic resonance imaging at the current presentation (a) Sagittal T2-weighted image of the cervical spine, (b) Sagittal T2-weighted image of the lumbar spine, (c)Sagittal T2-weighted image of the whole spine. The arrowhead shows an intramedullary T2-hyperintense lesion representing a syrinx, extending from the foramen magnum to T10 and showing marked longitudinal extension compared with the MRI obtained 21 years before.

**Figure 4:**
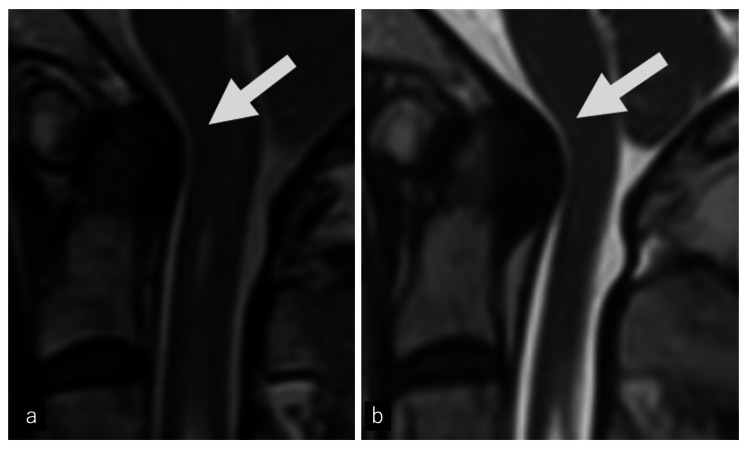
Serial cervical spine magnetic resonance imaging (MRI) 21 years before presentation and at the current presentation (a) Sagittal T2-weighted image 21 years before presentation, (b) Sagittal T2-weighted image at the current presentation. The arrow indicates a retro-odontoid pseudotumor, seen as a newly appeared T2-hypointense mass posterior to the odontoid process on comparison of the current MRI with that obtained 21 years before.

MRI revealed cerebellar tonsillar descent below the foramen magnum, loss of CSF space at the craniovertebral junction, a ROP compressing the ventral medulla, and a syrinx extending from the foramen magnum to T10 (Figures 3, 4).

The ROP appeared isointense on T1, hypointense on T2, and non-enhancing, consistent with a fibrocartilaginous lesion. No laboratory evidence of inflammatory disease was found.

FMD was performed. A 3 × 3 cm portion of the occipital bone was removed inferior to the external occipital protuberance, followed by C1 laminectomy and partial laminectomy of C2. The dura was thinned and adherent to the bone. The dura was opened while preserving the arachnoid membrane, and duraplasty was completed using both inlay and onlay DuraGen® (Integra LifeSciences, Princeton, USA) without suturing. Because preoperative imaging revealed no atlantoaxial instability, fixation was not performed.

**Figure 5:**
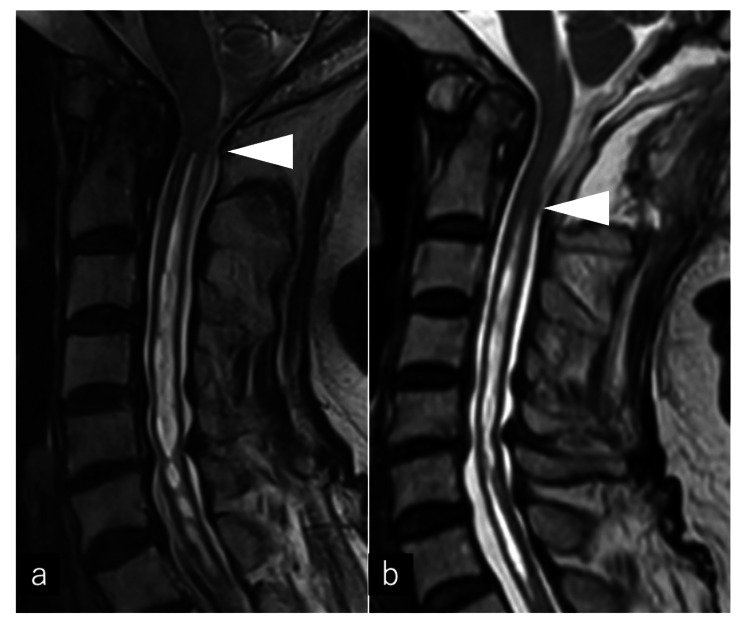
Comparison of preoperative and postoperative cervical spine magnetic resonance imaging (a) Preoperative sagittal T2-weighted image, (b) Postoperative sagittal T2-weighted image. The arrowhead indicates an intramedullary T2-hyperintense lesion representing a syrinx, which regressed postoperatively from the foramen magnum level to the C2 level.

The postoperative course was uneventful, with no complications. Within weeks, upper limb numbness improved to 2 on the NRS score, and hand pain completely resolved to 0 on the NRS score. Postoperative cervical spine MRI demonstrated a reduction of syrinx (Figure 5).

After approximately one month of rehabilitation, she was discharged home, independent in activities of daily living (ADL). At the two-year follow-up, she remained neurologically stable and was able to walk independently. Muscle strength in all limbs improved, with the ASIA motor score reaching UEMS 48/50 and LEMS 50/50. Only slight fingertip numbness persisted, with numbness severity rated 1 on the NRS score, whereas pain remained 0 on the NRS score.

## Discussion

CM-I is most commonly diagnosed between the late 20s and early 30s, with recent studies reporting a median age of approximately 40 years for adult patients [2,9]. This trend toward older diagnosis likely reflects the widespread availability of magnetic resonance imaging. Among adults diagnosed with CM-I, approximately 15-30% are asymptomatic, and conservative management is typically recommended for asymptomatic or mildly symptomatic cases [10]. While the long-term outcomes of conservative management have been well documented in pediatric cohorts, data for adults remain limited. Killeen et al. reported that only 25.5% of adults with symptomatic CM-I experienced symptom progression during conservative management, although the mean follow-up period was 4.9 years [11]. Longer-term observation could reveal higher rates of deterioration. Even in patients with long-term clinical stability, symptom exacerbation can occur later in life, underscoring the importance of continuous follow-up in elderly individuals, as exemplified by the present case. The patient remained neurologically stable for nearly two decades after diagnosis but experienced progressive deterioration in her eighties, ultimately necessitating surgical intervention.

In elderly patients, age-related changes may further modify the pathophysiological mechanisms of CM-I. The progression of symptoms involves both mechanical compression at the craniovertebral junction and impaired CSF circulation [12]. The myodural bridge complex (MDBC), which connects the suboccipital muscles to the dura mater, contributes to CSF dynamics through a pump-like mechanism [13-15]. Age-related loss of elasticity in this structure may reduce its efficiency, leading to CSF stagnation and worsening symptoms.

ROPs are typically associated with atlantoaxial instability or rheumatoid arthritis, but they may also occur in elderly patients without instability due to chronic mechanical stress [6]. In the present case, age-related degenerative changes and the appearance of ROP may have collectively contributed to the neurological deterioration associated with CM-I.

To better contextualize surgical outcomes in this age group, Table 1 summarizes reported surgical outcomes of CM-I in patients aged 65 years or older [16-19].

**Table 1:** Summary of surgical treatment for Chiari malformation type I in elderly patients

Nr.	Author, Year	Age/Sex	Surgery			Outcome	Follow-up	Notes
			Bone	Dura mater	Vertebral arch			
1	Wada et al. [15]	75/F	Suboccipital craniectomy	Dural plasty Goretex	C1 laminectomy C2 partial	Improved	5 months	none
									2	Takigami et al. [16]	69/F	Suboccipital craniectomy	Dural splitting	C1 laminectomy	Improved	24 months	Syringomyelia
3	Satake et al. [17]	75/F	Suboccipital craniectomy	Dural splitting	C1 laminectomy	Improved	3 months	Hydrocephalus
4	Badary et al. [18]	65/F	Suboccipital craniectomy	Dural plasty	C1 laminectomy	Required reoperation	125 months	Hydrocephalus, Syringomyelia
									5	Badary et al. [18]	72/F	Suboccipital craniectomy	Dural splitting	C1 laminectomy	Improved	59 months	Syringomyelia
6	Badary et al. [18]	78/M	Suboccipital craniectomy	Dural plasty	C1 laminectomy	Improved	348 months	Syringomyelia
									7	Badary et al. [18]	69/F	Suboccipital craniectomy	Dural plasty	C1 laminectomy	Died (unrelated cause)	14 months	Syringomyelia
8	Present case	82/F	Suboccipital craniectomy	Dural plasty DuraGen	C1 laminectomy C2 partial	Improved	24 months	Syringomyelia, Retro-odontoid pseudotumor

Surgical management in elderly patients remains a topic of debate due to concerns about bone fragility, comorbidities, and limited clinical experience. Whereas surgical treatment for younger patients with CM-I is often primarily directed toward decompression, elderly patients are more likely to have instability at the craniovertebral junction; therefore, the appropriate extent of decompression and the potential need for additional fusion should be considered more carefully. Nevertheless, prior studies have demonstrated that FMD can yield favorable outcomes when carefully selected. The present case represents one of the oldest reported patients (82 years) who underwent FMD with C1 laminectomy and partial C2 laminectomy, resulting in sustained neurological and radiological improvement over two years. These findings suggest that age alone should not preclude surgical consideration when progressive neurological deterioration occurs, provided that careful perioperative assessment and minimally invasive techniques are employed.

Finally, from a technical standpoint, duraplasty remains an essential component of FMD when CSF flow obstruction is identified. Techniques include dural incision with autologous fascia or synthetic grafts. Recent reports have found no significant difference in postoperative complications between sutured and sutureless duraplasty, though sutureless methods using DuraGen® reduce operative time and cost [20]. In the present case, marked dural thinning and strong adhesion to the bone made watertight suturing difficult; thus, inlay and onlay sutureless duraplasty using DuraGen® was performed. The postoperative course was uneventful, and neurological recovery remained sustained during follow-up. This case demonstrates that even in advanced age, FMD with adjunctive laminectomy and sutureless duraplasty can be safely and effectively performed, yielding durable clinical and radiological improvement.

## Conclusions

This case highlights that foramen magnum decompression with laminectomy can be a safe and effective treatment for elderly patients with CM-I complicated by ROP. Even after long-term clinical stability, neurological deterioration may occur in advanced age, emphasizing the need for ongoing follow-up. This report supports the notion that accurate diagnosis, timely surgical decision-making, and individualized decompression can result in durable long-term improvement in elderly patients with CM-I associated with ROP.
